# Intraoperative Fluorophores: An Update on 5-Aminolevulinic Acid and Sodium Fluorescein in Resection of Tumors of the Central Nervous System and Metastatic Lesions—A Systematic Review and Meta-Analysis

**DOI:** 10.3390/tomography9050124

**Published:** 2023-08-22

**Authors:** Sanjit Shah, Natalie Ivey, Abhijith Matur, Norberto Andaluz

**Affiliations:** University of Cincinnati Medical Center, Cincinnati, OH 45209, USA

**Keywords:** intraoperative fluorescence, fluorescein, aminolevulinic acid, central nervous system tumor, glioma

## Abstract

Introduction: Recent advances in tumor visualization have improved the extent of resection (EOR) of primary and secondary tumors of the central nervous system, while limiting the morbidity and mortality of the surgery. One area of recent interest has been the use of intraoperative fluorophores for tumor visualization such as 5-aminolevulinic acid (5-ala) and sodium fluorescein. We performed a systematic review and meta-analysis on the utility of fluorophore administration and EOR with each fluorophore to update the current literature. Methods: We conducted a systematic review and meta-analysis on the use of intraoperative 5-ala or fluorescein between 2021 and 2023 using the PubMed, SCOPUS, and WOS databases. The initial search yielded 8688 results. After inclusion and exclusion criteria were met, 44 studies remained for review. A meta-analysis was performed to compare the EOR between studies for each fluorophore and to compare the presence of intraoperative fluorescence by tumor type. Odds ratios (OR) were calculated for gross total resection (GTR), and two-way ANOVA tests were performed to compare rates of intraoperative fluorescence by fluorophore and tumor type. Results: In all groups except low-grade glioma, fluorescence was present after 5-ala administration; fluorescence was present for all groups after fluorescein administration. Two-way ANOVA analysis for both fluorophores demonstrated no statistically significant difference in presence of fluorescence between type of tumor resected. Meta-analysis of EOR did show a higher, but not significant, rate of GTR in the 5-ala group compared to controls (OR = 1.29, 95% CI = 0.49; 3.37). In the fluorescein group, there were statistically significant higher odds of GTR compared to the control group (OR = 2.10, 95% CI = 1.43; 3.10, I^2^ = 0%). Conclusions: Both 5-ala and sodium fluorescein demonstrated intraoperative fluorescence among various tumor types in both cranial and spinal tumors, as well as efficacy in improving EOR. Both fluorophores merit further investigation for use in surgery of CNS tumors.

## 1. Introduction/Background

Primary and secondary central nervous system tumors are a frequent source of morbidity and mortality in the population, with an estimated 6.2 new cases per 100,000 people annually in the U.S and a mortality rate of 4.4 per 100,000 people annually [1]. Perhaps more importantly, primary and metastatic central nervous system (CNS) tumors represent a significant source of morbidity, with neurologic symptoms such as weakness, aphasia, and cranial nerve dysfunction resulting from tumor location, degree of parenchymal invasion, and perilesional edema. The standard of care for most CNS lesions, primary or metastatic, typically involves an interdisciplinary approach that consists of a combination of resection, chemotherapy, and radiation therapy, though this varies based on tumor type. In glioblastoma multiforme (GBM), the most common primary malignant intracranial tumor, standard of care is maximally safe resection and subsequent radiotherapy, which has been shown to have significantly improved survival compared to surgery or radiation alone [2]. Conversely, benign tumors, such as typical meningiomas, can often be either observed or definitively cured with resection alone. Ultimately, surgical resection plays a central role in the resection of nervous system tumors.

Recent advances in surgical technique and adjunctive measures have improved the extent of resection (EOR) in patients with primary or metastatic CNS tumors. Though most of these advances have been noted in the resection of high-grade gliomas (HGG) and GBM, more recent advances are being made across the spectrum of tumor types. The utilization of fluorophores intraoperatively, sodium fluorescein and 5-aminolevulinic acid (5-ALA) in particular, has shown promise in improving EOR and is an area of significant focus for researchers.

### 1.1. 5-Aminolevulinic Acid

5-ALA (Gleolan^®^, Medexus Pharmaceuticals, Inc., Bolton, ON, Canada) is currently the only fluorophore with regulatory approval for use in high-grade glioma surgery in the United States [3,4]. First studied in 1980, it has been used in over 40 clinical trials and obtained regulatory approval in 2017 for standard of care in high-grade gliomas, offering a survival advantage of 6.2 months with some studies citing a specificity and sensitivity of 100% and 85%, respectively [3]. After being administered orally in the preoperative period, the drug demonstrates intracellular uptake and is metabolized secondary to blood–brain barrier disruption in diseased tissue. The oral bioavailability of 5-ALA is estimated to be 60% with a half-life of 45 min, and once absorbed, it is metabolized in the heme biosynthesis pathway in the mitochondria. Ferrochelatase converts 5-ALA to protoporphyrin, which fluoresces in the tumor’s intracellular space. It emits a violet-red fluorescence (640 to 710 nm) once activated with 375–440 nm light. The molecule is then effluxed by the ABCG2 pump into the blood, where it is excreted in the bile. The protoporphyrin accumulation is dependent on its efflux, biosynthesis, and protoporphyrin conversion to heme [5,6,7,8].

In general, Gleolan is given 2 to 4 h prior to anesthesia, since peak fluorescence occurs at six hours, aligning with the operative timing of dural incision [5,6,7,8]. Prolonged exposure to light can confer a photobleaching effect, minimizing the effectiveness of 5-ala. Fluorescence, although weak, can persist up to eight to nine hours after injection and include other areas of the CNS with diminished BBB, such as ependyma, choroid plexus, arcuate nucleus, median eminence, and areas with BBB breakdown secondary to malignancy [5,6,7,8]. The adverse events reported with Gleolan include skin sensitivity and clinically insignificant elevations in liver enzymes, with major adverse effects rarely cited in the literature [3].

### 1.2. Sodium Fluorescein

Fluorescein-sodium (C_20_H_12_O_5_-Na) is a water-soluble, orange-red powder that weighs 376 Da and has been widely utilized in the scientific and medical community. Its various applications include fluorescein isothiocyanate 1 (FITC) and the Alexa 488 fluorophore in cellular imaging, among others. The use of fluorescein for CNS tumors was first described by Moore and colleagues in 1947 [9]. The fluorescein biomarker is excited by a wavelength of 460 to 500 nm, and emits fluorescence in the 540 to 690 nm range [4,10]. Though fluorescein administration in for resection of CNS tumors is not currently FDA-approved, it has a wide breadth of ophthalmologic applications, and ongoing clinical studies are evaluating its use in CNS tumor resection.

Fluorescein’s intracranial mechanism of action relies on the disruption of the normal blood–brain barrier. The typical BBB is characterized by tight junctions between endothelial cells and astrocytic end feet separated by a basal laminar layer, responsible for maintaining the immunoprivileged nature of the CNS. It has been widely shown that tumors disrupt the BBB, allowing for leakage of fluids into the extracellular space. In this manner, fluorescein accumulates in the extracellular space and has been shown to correlate with gadolinium enhancement of glioma boundaries on MRI [11]. Importantly, this implies that fluorescein is not specifically targeting tumor cells, but rather targets areas with disrupted BBB [5,6,10]. Still, histopathologic analysis has demonstrated fluorescein administration to be highly specific in delineating tumor boundaries, with one study citing specificity at 90.9% and sensitivity at 82.2% [11]; these findings have since been corroborated by other studies [12,13,14,15,16].

Administration of fluorescein is typically performed ten to twenty minutes prior to incision in the dura. The majority of unbound fluorescein diffuses into the tumor while the remaining 30% of fluorescein remains in circulation, binds to albumin, and contributes to peak fluorescence. It fluoresces at 460 to 500 nm of light, and its characteristic yellow-green peaks at thirty minutes and last approximately four hours in the extracellular space. In addition, dura, the choroid plexus, and other circumventricular organs can also fluoresce. It ultimately washes out of the CNS circulation via renal excretion. The optimal dosing and timing of fluorescein administration to maximize tumor tagging and minimize fluorescence of normal brain tissue remains controversial [5,6], but reports of intravenous dosing for tumor resection vary from 3 mg/kg to 20 mg/kg. The more recent, lower dosing utilizes special operative microscopes with excitation and observation filters, while higher dosing is visible to the naked eye [17,18,19,20]. The administration of fluorescein has been shown to be typically well tolerated with minimal side effects. However, there have been two reported cases of anaphylaxis when used for resection of gliomas, both of which were treated in the ICU and resolved with supportive management [18,21,22].

## 2. Objectives

We performed a systematic review and meta-analysis on more recent literature surrounding 5-ala and sodium fluorescein to better understand the role of these fluorophores in resection of CNS tumors. The outcomes of interest were the presence or absence of fluorescence intraoperatively, and the overall EOR. A secondary outcome measured was the presence or absence of adverse effects.

## 3. Methods

### 3.1. Literature Search

A systematic review was conducted using the Preferred Reporting Items for Systematic Reviews and Meta-Analyses (PRISMA) guidelines [23]. PubMed, SCOPUS, and Web of Science were searched for English-language studies from January 2021 to March 2023, according to the following search terms for any field of the text: [(Fluorescein) AND (Glioma OR Brain Tumor OR Spinal OR Metastasis OR Tumor)] OR [{(5-Aminolevulinic Acid OR 5-ALA OR Gleolan) AND (Glioma OR Brain Tumor OR Spinal OR Metastasis OR Tumor)}] PubMed, SCOPUS, and Web of Science citations were imported into Rayyan.ai to remove duplicates and facilitate study selection. January 2021 to March 2023 was chosen as the study period to update the existing literature as several systematic reviews examine fluorophore use prior to January 2021, but very few have included data since 2021. 

### 3.2. Study Selection

A priori inclusion and exclusion criteria were set using the PICOS framework. Randomized controlled trials (RCTs), retrospective cohort studies, and case series that included more than three patients were included. Only studies that included the use of 5-ala or sodium fluorescein were included for analysis. Studies were screened for the primary outcomes, which were inclusion about data regarding the presence or absence of intraoperative fluorescence after administration of the selected fluorophore, or extent of resection after fluorophore administration. Two investigators (S.S. and N.I.) independently reviewed and screened each abstract for inclusion in full-text review. Discrepancies between reviewers were resolved by consensus; if no consensus could be reached, the most senior author (N.A.) decided whether to include the study (however, no studies required this step). Upon full-text review, studies were excluded if they did not use the correct fluorophore, did not provide information about intraoperative fluorescence, included the wrong population, or included the wrong outcome. Studies were evaluated for bias using the JBI critical appraisal tool by S.S. [24].

### 3.3. Data Extraction

One reviewer (S.S.) extracted data from each article, which was then confirmed independently by one additional reviewer (N.I.). Missing data were not reported by the authors. Data included: author, study design, sample size, journal, fluorophore administered, presence of intraoperative fluorescence, extent of resection, safety events, and survival data. Presence of intraoperative fluorescence was sorted by type of tumor included in each respective study. Extent of resection was sorted by gross total resection (GTR) or subtotal resection (STR). Several studies included a category for near total resection, but the definition of near total resection was inconsistent, so all near total resections were included as subtotal resection.

### 3.4. Statistical Analysis

Meta-analysis was performed using R version 4.1.0 (The R Foundation for Statistical Computing, open source software). A two-tailed *p*-value < 0.05 was used to determine significance. When comparing extent of resection between studies, rates of gross total resection (GTR) as determined by each study were used for comparison. Dichotomous outcomes of presence of GTR and type of fluorophore administered were pooled using the Mantel–Haenszel method, and the Paule–Mandel estimator was used for t2. A random-effects meta-analysis model was then used to give a pooled estimate of the outcome as an odds ratio (OR) for GTR. Two-way ANOVA tests were run when comparing the type of tumor resected and presence of intraoperative fluorescence. The random-effects model was chosen over a fixed-effects model for all study variables due to differences in study design, patient selection, and measurement of outcomes, which may result in significant variation between studies not due to chance. 

## 4. Results

### 4.1. Review Characteristics

**Figure 1:**
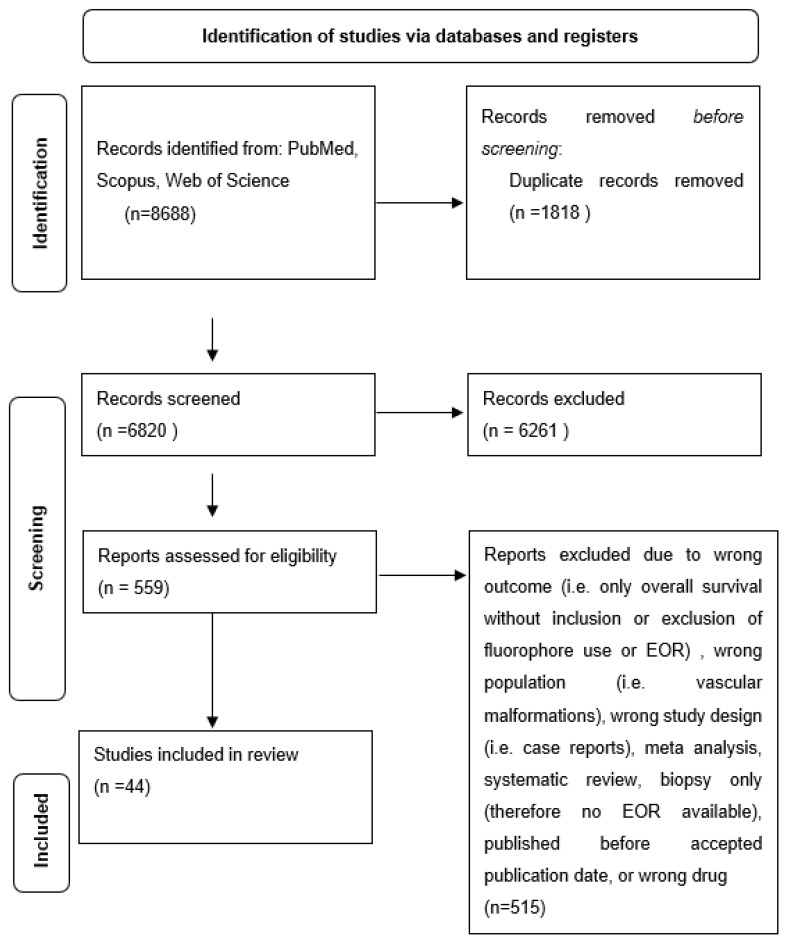
Flow diagram of included studies in review.

After all records had been screened for eligibility, the remaining studies were analyzed for study type, population type, outcome type, and appropriate drug administration (Figure 1). Ultimately, 44 studies were included in the study. The studies ultimately included for analysis are outlined in Table 1. 

### 4.2. Intraoperative Fluorescence

**Figure 2:**
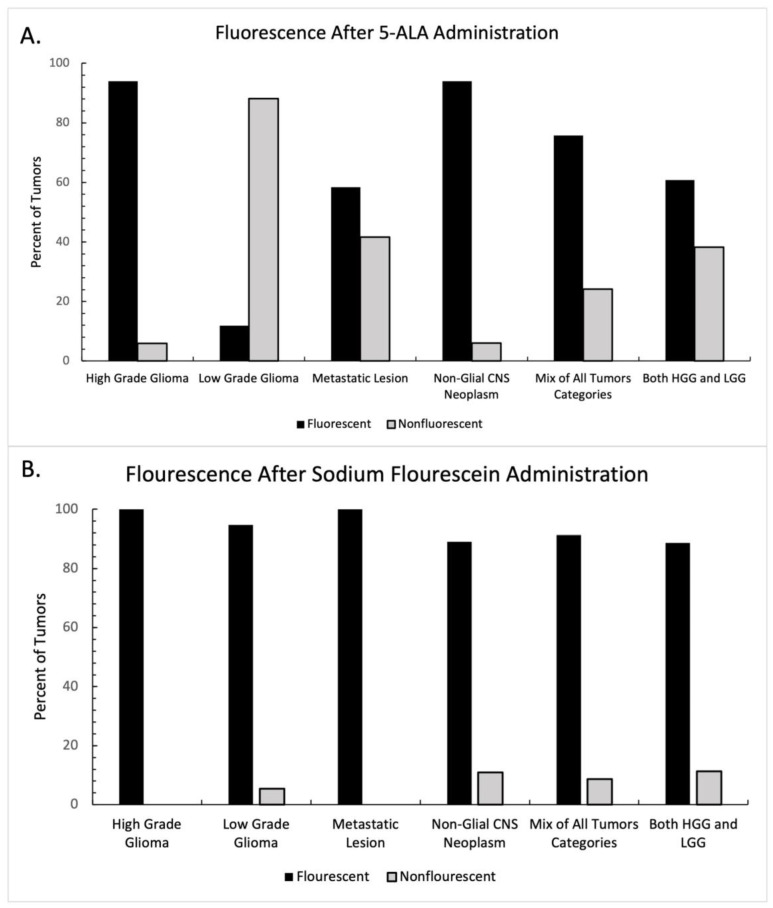
**Intraoperative fluorescence after fluorophore administration, distributed by tumor type. Percentage of tumors with intraoperative fluorescence after (A) 5-ala administration and (B) sodium fluorescein administration. 5-ala demonstrated heterogenous enhancement depending on tumor type, while sodium fluorescein demonstrated consistent intraoperative fluorescence across tumor** **type.**

The presence or absence of fluorescence was reported in thirty-five of the studies included in this systematic review, 20 in the 5-ala group and 15 in the fluorescein group. Each study was categorized according to the class of tumor being resected (Table 1). Tumors in the “mixed tumor” category and the “all glioma” category were excluded from statistical analysis, as the presence or absence of fluorescence was not regularly reported in the context of tumor type. The graphical breakdown of intraoperative fluorescence by tumor type is shown in Figure 2A. In the 5-ala group, administration of the fluorophore demonstrated intraoperative fluorescence more frequently than not in all groups except for low-grade gliomas, where intraoperative fluorescence was seen infrequently. Intraoperative fluorescence also demonstrated mixed results in the setting of metastatic disease with 5-ala. A two-way ANOVA was performed between tumor type and the presence of fluorescence, which did not show statistical significance (F = 2.67, *p* = 0.316). 

A similar analysis was performed for studies reporting sodium fluorescein use. The graphical breakdown of intraoperative fluorescence by tumor type is shown in Figure 2B. In all groups, fluorescence was present far more frequently in the sodium fluorescein group than not. A two-way ANOVA was again performed between tumor type and the presence of fluorescence, which did not show statistical significance (F = 0.266, *p* = 0.848).

### 4.3. Extent of Resection

**Figure 3:**
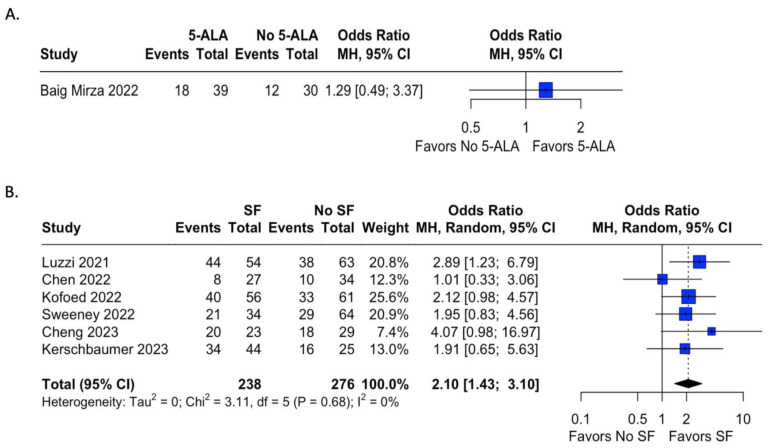
**Meta-analysis** **of extent of resection of tumor after fluorophore administration. Odds ratios were calculated for rates of GTR for studies reporting either 5-ala or fluorescein use and a control cohort. Analysis of 5-ala favored the use of 5-ala but did not reach statistical significance, while analysis of sodium fluorescein demonstrated statistically significant odds of achieving GTR with fluorescein administration compared to the control group with relatively low heterogeneity [15,49,57,71,72,79,85].**

Extent of resection was reported in 32 studies, 15 in which 5-ala was used, and 17 where fluorescein was used. Table 1 shows the extent of resection by study. Studies in which a control group was not present were excluded from analysis. In studies with a control group, odds ratios were calculated for the presence of gross total resection (GTR) by use of fluorophore. Only one study in which 5-ala was used during the time frame for this meta-analysis presented data on EOR for both an experimental and control group; results are demonstrated in Figure 3A and were not found to be statistically significant (OR = 1.29, 95% CI = 0.49; 3.37). In contrast, six studies using fluorescein included data on EOR in both groups with and without fluorophore use (Figure 3B). The use of sodium fluorescein was found to have statistically significantly higher odds of achieving GTR than in cases without fluorescein (OR = 2.10, 95% CI = 1.43; 3.10, I^2^ = 0%).

### 4.4. Fluorophore Safety

**Figure 4:**
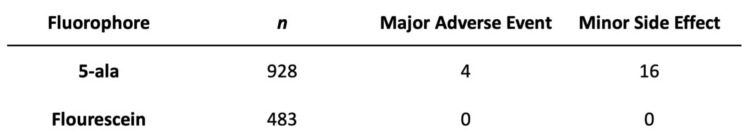
**Safety** **profile of fluorophores. Both fluorophores were generally well tolerated with minimal to no adverse events or side effects.**

In total, 27 studies commented on the safety of fluorophore use, 14 in the 5-ala group and 13 with fluorescein use. Figure 4 demonstrates the frequency of both major adverse events and minor side effects with use of each fluorophore. As noted, side effects were rare in both groups, with only four reported major adverse events with 5-ala use, and only sixteen cases of minor side effects. There were no major or minor adverse effects reported with the use of sodium fluorescein. 

## 5. Discussion

The use of intraoperative fluorophores in tumor resection has become increasingly frequent, and with improving technology, has led to improvements in extent of tumor resection. In this study, we aimed to assess the utility of fluorophore administration, its impact on extent of resection, and the safety of each fluorophore in studies conducted during the past three years. Our approach was unique as it included all CNS tumors, regardless of location (cranial or spine) or tumor type. Largely, our findings support the use of either fluorophore during resection of CNS tumors, though in the case of 5-ala, utility in low-grade gliomas and metastatic disease requires further evaluation. 

In our study, the use of either 5-ala or sodium fluorescein largely demonstrated intraoperative fluorescence of the tumor and tumor boundaries. The use of 5-ala in high-grade gliomas was particularly useful, but as reported in the literature, use of 5-ala in low-grade gliomas was of limited utility and demonstrated mixed results in metastatic disease. In 2015, Jaber and colleagues noted that out of 82 low-grade gliomas, only 13 demonstrated intraoperative fluorescence with 5-ala [25]. Similarly, Hossman et al. found that only 7 out of 59 low-grade gliomas demonstrated intraoperative fluorescence with 5-ala; further analysis by this group noted that fluorescence was focal and limited to more aggressive areas of the tumor [26]. Other groups have reported similar rates of low intraoperative fluorescence of LGG with 5-ala [27,28,29]. Our study found that detection of metastatic lesions with 5-ala was heterogenous, again in line with the published literature. Mercea and colleagues reported a 69% rate of fluorescence in one cohort, while Marhold reported only 28% fluorescence in another recent study [30,31]. Previously published cohort studies report similar findings, with fluorescence noted to be heterogenous with regard to tumor boundaries [32,33,34]. Bettag and colleagues noted that although there was only 57% fluorescence under a standard white light microscope, fluorescence in the same group of tumors approached 84% with the use of an endoscope [35]. This underscores the value that advances in adjunct technologies might have on improving the role of intraoperative fluorophore use in surgery. Our study corroborates previously extensively reported evidence that 5-ala fluorescence is particularly useful in high-grade glioma surgery [36,37,38]. Notably, our study also found similar utility of 5-ala in identifying other CNS neoplasms (pleiomorphic xanthastrocytomas, oligodendrogliomas, etc.) [39,40]. Though our analysis did not reach statistical significance when comparing the rate of fluorescence by tumor type, we did observe that LGG demonstrated remarkably different fluorescence rates and patterns to other CNS tumors. 

The use of sodium fluorescein in our study, by contrast, showed far more homogenous results. Regardless of tumor type, sodium fluorescein demonstrated no significant difference in rate of fluorescence. Out of all studies included in our analysis, all demonstrated fluorescence rates above 77%, and 11 out of 15 demonstrated fluorescence rates > 90% [41,42,43,44,45,46,47,48,49,50,51,52,53,54,55]. Previous prospective cohort studies by Acerbi have shown a high specificity and sensitivity of fluorescein in glioma detection, citing numbers as high as 79.1% and 80.8%, respectively [12]. Diaz et al. reported even higher rates of sensitivity and specificity at 82.2% and 90.9%, respectively [11]. One study showed 88% homogenous and the remaining 12% heterogenous fluorescence of meningiomas [56]. Part of the reason that intraoperative fluorescence was higher across tumor groups in the fluorescein cohort may be that, unlike 5-ala, fluorescein is not dependent on an intracellular metabolic process for activation, but rather accumulates in the extracellular space at areas of BBB breakdown.

Extent of resection was also evaluated in our meta-analysis. In the 5-ala group, only one study that reported EOR also reported the results of a control group [57]. Though the odds of GTR did not reach statistical significance in this study, it is worth noting that multiple studies have published their EOR using 5-ala, with consistently improved odds of GTR or improved EOR when using 5-ala [36,38,58]. As noted in Table 1, rates of GTR in 5-ala cohorts without a control group were variable. This suggests that further investigation into the rate of GTR using 5-ala is warranted, especially when considering resection of CNS tumors that do not fall into the HGG category. By contrast, six studies in the fluorescein group reported statistics for both EOR in the fluorescein group and the control group. The odds of GTR with fluorescein were shown to be significantly higher than those in the control groups; moreover, the studies showed little heterogeneity in outcome and no single study unduly influenced the outcome. Notably, unlike the 5-ala group, these studies were variable in the type of tumor being resected; these results provide convincing evidence that the use of intraoperative fluorescein does improve the odds of GTR. As early as 2008, Koc et al. showed a GTR rate of 83% with fluorescein compared to 55% in the control group [14]. In the prospective cohort FLUGLIO Phase II study, Acerbi found that fluorescein was effective with a GTR rate of 82.6% [12]. While Table 1 shows a trend toward generally favorable rates of GTR with fluorescein in all tumor types, more stringent studies will be required to better characterize this effect. Ultimately, both 5-ala and fluorescein demonstrate promising results with regard to the extent of tumor resection.

Safety data for both fluorescein and 5-ala were assessed during the study. In our review, we found no adverse reactions associated with fluorescein use, and a limited number of severe adverse events and side effects associated with 5-ala use. With regard to fluorescein, the dye has been widely applied in the field of ophthalmology, with side effects being fairly infrequent [59,60]. At least two case reports of anaphylaxis after fluorescein administration for CNS tumor surgery have been reported, but in both the dosing was 20 mg/kg, the highest dose reported for this administration [18,22]. Restelli and colleagues reported a patient who erroneously received 30 mg/kg of fluorescein without any adverse effects [21]. The safety of 5-ala has been well documented, including in large randomized controlled trials (RCTs) with no significant difference between the rate of adverse events in groups treated with 5-ala compared to control groups [37]. Both 5-ala and fluorescein appear safe for clinical use with regard to improving intraoperative visualization. 

Of note, one unique characteristic of our study is the breadth of tumor cohorts we included. Unlike many studies, we included data from both pediatric and adult patients, as well as cranial and spine tumors, and all CNS tumor types, both primary and secondary. The inclusiveness of this study was deliberate, as our goal was to understand how the broad spectrum of CNS tumors behaved with regard to 5-ala and fluorescein administration, and whether EOR was improved. The results presented above do suggest that 5-ala differentially fluoresces for HGG compared to LGG and metastatic lesions, but the same does not hold true for fluorescein. Similar conclusions can be drawn for EOR, as in the lone 5-ala study the sole tumor type was HGG, while in the six fluorescein studies in which EOR was analyzed, tumor type varied from HGG to metastatic lesion to other non-glial CNS primary malignancy.

One major limitation of our study is that we did not report overall survival (OS) or progression-free survival (PFS) as an outcome. This was in part due to the heterogenous nature in which survival has been reported throughout the literature, obscuring the ability of statistical analyses to be performed. A 2021 retrospective cohort study by Baig Mirza et al. showed improved OS, EOR, and performance status in GBM with 5-ala-assisted surgery when compared to the control group; subgroup analysis showed an even greater OS benefit and performance status improvement in patients undergoing GTR [58]. Though Stummer and Eljamel both showed a significant improvement in PFS with 5-ala use during early RCTs on 5-ala use [36,37], a 2017 systematic review showed varying results for OS between studies, fluctuating between significance and non-significance [61]. Multiple cohort studies have shown promising data on OS or PFS for GBM, but have been heterogenous in their approach and without a control group to provide Level 1 evidence [38,39,61,62,63,64]. In another recent study regarding Grade III tumor resection, OS was not significantly improved in 5-ala-assisted surgery compared to the control groups; however, a subgroup analysis showed that patients with 5-ala-guided resection who underwent GTR showed a highly significant improvement in OS compared to the control group [57]. Hosmann and colleagues noted that, in patients with LGG, fluorescence of 5-ala was associated with shorter PFS (2.3 ± 0.7 vs. 5.0 ± 0.4 years; *p* = 0.01), suggesting that fluorescence may portend a more aggressive tumor and thus worse prognosis [26]. When evaluating non-glial CNS tumors or metastatic lesions, survival data for 5-ala guided resection was sparse. 

One additional limitation of our study was that individual studies did not include homogenous criteria for what constituted gross total resection. Indeed, resection of ring enhancing lesion in GBM might be easier to identify radiologically than resection of low-grade gliomas, which often are poorly defined radiologically. As such, one weakness of our study is the underlying assumption that GTR was generally assessed in a similar fashion by radiologists and neurosurgeons across the included studies. Similarly, each individual study had a heterogenous set of inclusion and exclusion criteria, leaving the systematic review and meta-analysis vulnerable to selection bias based on the published studies included. In addition, publication bias would favor positive results with the use of both fluorescein and 5-ALA, again potentially biasing the results of this study. Similarly, confounding factors such as demographic factors of patients, comorbidities, and surgeon experience were unable to be controlled for given the nature of our review, again exposing the study to confounding variables. 

It is also possible that other drugs routinely administered in the treatment of CNS tumors, such as antiepileptics and steroids, may impact the efficacy of fluorophores. One recent study by Goryaynov and colleagues noted a negative correlation between antiepileptic administration and intraoperative fluorescence, with 73% of patients treated with antiepileptic medications prior to surgery having no visible 5-ALA fluorescence, in contrast to 83% of antiepileptic naïve patients demonstrating visible fluorescence [65]. A multivariate analysis of intraoperative 5-ALA fluorescence in 175 IDH-1 wildtype GBM patients revealed levetiracetam use as the only significant risk factor for reduced fluorescence [66]. These findings are corroborated by in vitro studies testing protoporphyrin accumulation in glioma cell lines when treated with either dexamethasone or commonly used antiepileptics [67,68]. However, a recent large retrospective cohort series by Wadiura and colleagues yielded no significant impact of corticosteroids or antiepileptics on intraoperative fluorescence [69]. Ultimately, the effect of antiepileptic drugs and corticosteroids on intraoperative fluorescence requires further study. 

In fluorescein-guided resection, there are no current RCTs comparing resection with a control group. However, Schebesch et al. found significantly improved OS with the use of SF during multivariate analysis during a large retrospective cohort review, and Falco and colleagues showed similarly promising OS (16 months) with the use of fluorescein in HGG [43,70]. These findings align with early data provided by Acerbi in the large FLUGLIO Phase II trial, as well as other studies [12,13]. Data behind survival benefit in metastatic lesions is more controversial, as Cheng and Kofoed both showed statistically significant increases in OS in the fluorescein-assisted surgery group [44,71], while Chen and Kerschbaumer showed no significant difference with the use of fluorophore [45,72]. Again, survival data on other tumor types is sparse. In the largest study utilizing both fluorescein and 5-ala, there did appear to be a trend towards improved survival with concomitant use of both fluorophores [73]. Future studies will likely focus even more on survival, making it feasible to quantify the survival benefits of 5-ala and fluorescein use further.

Another source of bias in our study was limiting the study period to 2021–2023; as many of the 5-ala studies were performed before this period, comparative data on 5-ala EOR was limited, as noted in Figure 2A. We chose this period as most systematic reviews or meta-analyses on this topic include studies from before 2021, and as such exclude the more recent advances made using fluoresecein. However, a broader study period would likely yield a statistically significant OR with regard to 5-ala-assisted resection, as several RCTs have already shown improved EOR with 5-ala [36,38,58].

**Table 1:** List of papers included in systematic review and meta-analysis. In many studies, data on visualization, EOR, side effect profile, and survival benefit of fluorophore was incomplete. * Study was found to evaluate both 5-ALA and fluorescein; resections in which both were administered were excluded from our analysis but mentioned for survival benefit.

Author	Year	Type of Study	Fluorophore Used	Tumors Visualized (# of Visualization/Total Number Resected)	Type of Tumor	EOR with Fluorophore (#GTR/Total Number Resected)	EOR in Control Group (#GTR/Total Number Resected)	Adverse Events	Survival Benefit of Fluorophore
Alcazar et al. [46]	2023	Retrospective Case Series	Fluorescein	3/3	Other Non-Glial CNS Neoplasm (Various)	3/3	--	None	--
Baig Mirza et al. [57]	2022	Retrospective Cohort Study	5-ALA	28/39	HGG (Grade III Anaplastic Astrocytoma)	18/39	12/30	3 Major Adverse Events	No difference
Baig Mirza et al. [58]	2021	Retrospective Cohort Study	5-ALA	--	HGG (GBM)	120/253	--	--	Improved compared to non-5-ALA guided surgery
Batalov et al. [74]	2021	Retrospective Case Series	5-ALA	62/75	HGG & LGG	--	--	--	--
Bettag et al. [35]	2022	Retrospective Case Series	5-ALA	22/26	Metastatic Lesions	--	--	--	--
Cardali et al. [53]	2022	Retrospective Case Series	Fluorescein	13/14	Mixed Tumors (Various)	11/14	--	None	--
Certo et al. [39]	2021	Prospective Case Series	5-ALA	68/68	HGG (GBM/Gliosarcoma)	64/68	--	--	--
Chen et al. [50]	2022	Retrospective Cohort Study	Fluorescein	27/27	Other Non-Glial CNS Neoplasm (Medulloblastoma)	8/27	10/34	None	No difference
Cheng et al. [49]	2023	Retrospective Cohort Study	Fluorescein	23/23	Metastatic Lesions	20/23	18/29	None	Improved with Fluorescein-guided surgery
da Silva et al. [75]	2022	Retrospective Case Series	5-ALA	195/255	Mixed Tumors (Various)	--	--	1 Major Adverse Event	--
de Laurentis et al. [54]	2022	Retrospective Case Series	Fluorescein	35/45	Mixed Tumors (Various)	--	--	--	--
Falco et al. [48]	2023	Retrospective Case Series	Fluorescein	93/93	HGG (GBM)	77/93	--	None	--
Falco et al. [41]	2022	Retrospective Case Series	Fluorescein	41/44	LGG (Pilocytic Astrocytoma)	24/39	--	None	--
Falco et al. [42]	2022	Retrospective Case Series	Fluorescein	12/12	Other Non-Glial CNS Neoplasm (Pleiomorphic Xanthoastrocytoma)	8/12	--	None	--
Goryaynov et al. [76]	2022	Retrospective Case Series	5-ALA	20/34	HGG & LGG	--	--	None	--
Hohne et al. [55]	2021	Retrospective Case Series	Fluorescein	12/12	Mixed Tumors (Various)	--	--	None	--
Hosmann et al. [26]	2021	Retrospective Case Series	5-ALA	7/52	LGG	29/59	--	None	--
Ibrahim et al. [62]	2021	Retrospective Case Series	5-ALA	--	HGG & LGG	24/40	--	One Minor Side Effect	--
Kerschbaumer et al. [72]	2022	Retrospective Cohort Study	Fluorescein	--	Metastatic Lesions	34/44	16/25	--	No Difference
Kiesel et al. [64]	2021	Retrospective Case Series	5-ALA	161/163	HGG (GBM)	84/94	--	None	--
Kofoed et al. [71]	2022	Retrospective Case Series	Fluorescein	--	Metastatic Lesions	40/56	33/61	--	Associated With Increased Overall Survival
Kutlay et al. [43]	2021	Retrospective Case Series	Fluorescein	18/18	Mixed Tumors (Various)	16/18	--	None	--
Lavrador et al. [77]	2023	Retrospective Case Series	5-ALA	6/6	HGG	--	--	--	--
Luzzi et al. [78]	2021	Retrospective Cohort Study	Fluorescein	--	HGG (GBM + Anaplastic Astrocytoma)	44/54	38/63	--	Improved Progression Free Survival, Similar Overall Survival
Maragkos et al. [79]	2021	Retrospective Case Series	5-ALA	16/16	HGG	--	--	None	--
Marhold et al. [31]	2022	Retrospective Case Series	5-ALA	8/29	Metastatic Lesions	--	--	--	--
Mercea et al. [30]	2021	Retrospective Case Series	5-ALA	36/58	Metastatic Lesions	17/25	--	None	--
Millesi et al. [40]	2021	Retrospective Case Series	5-ALA	25/31	Other Non-Glial CNS Neoplasm (Ependymoma/Subependymoma)	25/31	--	None	--
Milos et al. [80]	2023	Prospective Case Series	5-ALA	5/14	Mixed Tumors (Various)	10/14	--	None	--
Muscas et al. [81]	2022	Retrospective Case Series	5-ALA	--	HGG (GBM)	41/65	--	--	--
Muther et al. [82]	2022	Retrospective Case Series	5-ALA	81/173	HGG & LGG	--	--	--	--
Olguner et al. [44]	2021	Retrospective Case Series	Fluorescein	47/49	Mixed Tumors (Various)	46/49	--	--	--
Ott et al. [51]	2022	Retrospective Case Series	Fluorescein	--	Mixed Tumors (Various)	11/14	--	None	--
Schebesch et al. [70]	2022	Retrospective Case Series	Fluorescein	--	HGG	--	--	None	--
Schupper et al. [63]	2022	Prospective Cohort Study	5-ALA	69/69	HGG	--	--	15 minor adverse events	--
Strickland et al. [83]	2022	Retrospective Case Series	5-ALA	24/30	HGG (GBM + Anaplastic Astrocytoma + Anaplastic Oligodendroglioma)	9/21	--	None	--
Sun et al. [52]	2022	Retrospective Case Series	Fluorescein	47/59	Other Non-Glial CNS Neoplasm (Ependymoma)	56/56	--	--	--
Sweeney et al. [84]	2022	Retrospective Cohort Study	Fluorescein	--	GBM	21/34	29/64	--	--
Takeda et al. [85]	2022	Retrospective Case Series	5-ALA	4/7	Mixed Tumors (Various)	6/7	--	None	--
Ung et al. [45]	2022	Retrospective Case Series	Fluorescein	12/12	Mixed Tumors (Various)	--	--	None	--
Watts et al. [86]	2023	Prospective Case Study	5-ALA	85/99	HGG (GBM)	75/99	--	--	--
Xue et al. [47]	2021	Retrospective Case Series	Fluorescein	44/50	HGG + LGG	41/50	--	--	--
Zeppa et al. * [73]	2022	Retrospective Case Series	5-ALA, Fluorescein	--	HGG	18/40 (5-ALA) 21/44 (Fluorescein)	--	--	Advantage after concomitant use
Zhang et al. [87]	2022	Retrospective Case Series	5-ALA	10/11	HGG	--	--	None	--

## 6. Conclusions

Both 5-ala and fluorescein appear to have utility in primary and secondary CNS tumor resection. In our review and analysis of the recent literature, both fluorophores appear to fluoresce in various tumor types. 5-ala may have limited utility in low-grade glioma, while fluorescein appears to more homogenously enhance intraoperatively, likely due to their respective mechanisms of action. While 5-ala shows a trend toward improving EOR, though limited due to our study period, fluorescein showed compelling evidence of improving rates of GTR regardless of tumor type and location. Both fluorophores were found to be generally safe, but careful selection of the fluorophore based on preoperative imaging, expected tumor type, and the patient’s current medications may better inform the surgeon which fluorophore to utilize. Further research on the use of both fluorophores will better elucidate their utility on the various types of CNS neoplasms.

## Data Availability

Data extracted from included studies, used for analysis, analytic code, and forms used for bias assessment are available for public access per request from corresponding author.
